# Therapeutic hypothermia for stroke: Unique challenges at the bedside

**DOI:** 10.3389/fneur.2022.951586

**Published:** 2022-10-03

**Authors:** Je Sung You, Jong Youl Kim, Midori A. Yenari

**Affiliations:** ^1^Department of Emergency Medicine, Yonsei University College of Medicine, Seoul, South Korea; ^2^Department of Anatomy, Yonsei University College of Medicine, Seoul, South Korea; ^3^Department of Neurology, The San Francisco Veterans Affairs Medical Center, University of California, San Francisco, San Francisco, CA, United States

**Keywords:** ischemic stroke, systemic therapeutic hypothermia, selective brain cooling, targeted temperature management, cell death, inflammation, clinical outcomes

## Abstract

Therapeutic hypothermia has shown promise as a means to improving neurological outcomes at several neurological conditions. At the clinical level, it has been shown to improve outcomes in comatose survivors of cardiac arrest and in neonatal hypoxic ischemic encephalopathy, but has yet to be convincingly demonstrated in stroke. While numerous preclinical studies have shown benefit in stroke models, translating this to the clinical level has proven challenging. Major obstacles include cooling patients with typical stroke who are awake and breathing spontaneously but often have significant comorbidities. Solutions around these problems include selective brain cooling and cooling to lesser depths or avoiding hyperthermia. This review will cover the mechanisms of protection by therapeutic hypothermia, as well as recent progress made in selective brain cooling and the neuroprotective effects of only slightly lowering brain temperature. Therapeutic hypothermia for stroke has been shown to be feasible, but has yet to be definitively proven effective. There is clearly much work to be undertaken in this area.

## Introduction

Acute ischemic stroke is the primary cause of about 85% of strokes worldwide and is most frequently caused by blood clots or atherosclerosis occluding cerebral blood flow (1, 2). Studies have shown that decreasing the time between presentation and intervention can improve clinical outcomes (2, 3). Modern management efforts thus emphasize the time sensitivity of stroke treatment. Antiplatelet and anticoagulant drugs have long been the mainstay of ischemic stroke management, along with the use of acute thrombolysis and mechanical thrombectomy to revascularize thrombosed vessels (4–6). If initiated rapidly, these revascularization strategies reduce morbidity and improve neurological outcomes, although the window for these interventions is on the order of hours (6, 7). Unfortunately, due to a variety of constraints, a majority of patients tend to present too late for these interventions and are not eligible for potentially life-saving and disability-preventing treatments (8–11). Thus, adjunctive treatments may be needed to extend this critical time interval to offer treatments to a broader number of stroke victims.

Therapeutic hypothermia (TH) has been suggested as a potential approach to achieve the goal (12, 13). In 2002, two randomized controlled trials (RCT) showed the induction of mild hypothermia (32–34°C) produced more favorable neurologic outcomes and improved survival after cardiac arrest compared with patients for whom body temperatures were maintained in the normothermic range (14, 15). TH has since been rapidly implemented worldwide and established as a gold standard in the management of comatose survivors of cardiac arrest; however, TH has only been shown to be effective at the clinical level in patients suffering from cardiac arrest and in neonates suffering from hypoxic ischemic encephalopathy (HIE) (16–18). In this review, TH refers to cooling the body in order to preserve organ viability and is so far the most effective therapy for improving neurological outcomes in comatose survivors of cardiac arrest (14, 18, 19). Subsequent studies have also shown that modulation of body temperature to normal and slightly below normal levels may also be beneficial. Thus, the term targeted temperature management (TTM) refers to modulation of body temperature including TH.

In spite of the optimism of TTM in cardiac arrest and neonatal HIE, clinical applications of TTM in other acute brain injuries, such as hemorrhagic or ischemic stroke and traumatic brain injury, have yet to demonstrate improvement in clinical outcomes (20–25). Yet, multiple preclinical studies have consistently shown that TH induces multiple and synergistic effects for neuroprotection in experimental models (16, 26). A major challenge in translating this to the clinical level is that therapeutic hypothermia studies in many experimental models used small species where whole body cooling can be achieved in a short period of time. By contrast, humans have much larger mass, and whole body cooling to achieve target brain temperatures for optimal neuroprotection requires many hours. Furthermore, patients with stroke are typically older with comorbidities, which could complicate TH. Thus, selectively cooling the brain has the potential not only to achieve more rapid cooling but may reduce systemic complications. Efforts to achieve this included using internal catheters to reduce temperatures of the cerebral vessels, which would then cool brain tissue (23, 25), or cooling caps to directly cool the brain (27–29), but these approaches have achieved limited success. Regardless, it is clear that reducing brain temperature can improve neurological outcomes from many acute brain insults. This review covers the current state of TTM as it relates to clinical stroke care.

## Mechanisms of hypothermic protection in experimental ischemic stroke

To understand the robust neuroprotective effects of TH, it is important to understand the preclinical works related to understanding why TH seems so effective. TH has long been thought to lead to beneficial effects by decreasing brain metabolism (16), but through multiple experimental studies, it has now been recognized that TH exerts neuroprotection by favorably altering a broad range of pathological pathways, including the regulation of brain metabolism, apoptosis, microglial activation, cerebral blood flow, inflammation, and neurotrophic factors (16, 26). As such, this may be a major reason why lowering brain temperature may lead to preservation of brain tissue and function.

### Effect of hypothermia on brain metabolism, blood flow, and excitotoxicity

In ischemic stroke models, hypothermia to brain at temperatures of 33°C (mild hypothermia) showed improved cerebral blood flow and preservation of the cellular metabolic rate (30, 31). During stroke, cerebral blood flow (CBF) is disrupted following vessel occlusion. If blood flow is restored (reperfusion), there is a brief and abrupt overshoot of CBF, followed by gradual deterioration. Microvascular narrowing was thought to underlie this deterioration (32), and TH has been shown not only to improve and maintain CBF by preventing microcirculatory collapse but also seems to prevent this brief overshoot of CBF upon reperfusion (33).

Brain metabolism is also sensitive to temperature. Mild hypothermia reduces oxygen consumption by a ~5%/°C decrease in body temperature in the range of 22–37°C (2). Cerebral ischemia also leads to increased accumulation of extracellular glutamate and influx of calcium (34). Hypothermia has been documented to prevent glutamate accumulation and subsequent excitotoxicity mediated by calcium influx (26, 35). More recently, hypothermia also appears to suppress the calcium-sensing receptor (CaSR) expression, which regulates calcium influx and upregulates the inhibitory gamma-aminobutyric acid B receptor 1 (GABA-B-R1) (36). As such, hypothermia appears to induce neuroprotective effects in ischemia models by affecting multiple aspects of brain metabolism and neurotransmission.

### Neuroprotection by hypothermia: Cell death pathways

Beyond early observations that hypothermia preserves tissue metabolic reserves and reduces ischemic elaboration of excitotoxins, hypothermia has also been shown to positively influence several ischemic cell death pathways, such as apoptosis (37). Several studies have shown that hypothermia can prevent apoptotic cell death in stroke models (38–40). TH was first shown to affect several aspects of the intrinsic pathway, ultimately leading to neuroprotection. The intrinsic pathway is initiated within the cell mitochondria *via* release of various factors, such as cytochrome c and apoptosis-initiating factor (AIF), into the cytosol (41). Mild hypothermia has been shown to increase the anti-apoptotic protein Bcl-2, which, in turn, inhibits cytosolic cytochrome c release and subsequent caspase activation (42). A few studies have also shown that mild hypothermia reduces the generation of pro-apoptotic Bax (43–46). Downstream of Bcl2, hypothermia has been shown to influence protein kinase C (PKC) family members, such as anti-apoptotic (PKCε) or pro-apoptotic (PKCδ), so as to lead to overreduction in apoptotic cell death (39).

The extrinsic apoptotic pathway is triggered *via* death receptors, which, when ligated, leads to cell death. A prototypical death receptor, Fas, and its corresponding ligand, Fas ligand (FasL), have also been studied in stroke models, and interrupting this pathway has been shown to improve outcomes in stroke models (47). TH has been shown to decrease the expression of Fas and FasL and subsequent activation of downstream caspase-8 (48–51).

TH can also affect caspase-independent apoptosis. The mitochondrial apoptosis-inducing factor (AIF) pathway involves direct apoptotic cell death and is capable of inducing apoptosis without activating caspases (52). Mild hypothermia suppressed AIF translocation from the mitochondria to the cytosol and led to reduced apoptotic cell death in an ischemic stroke model (53). In sum, TH is capable of influencing many cell death pathways in such a way so as to favor cell survival.

### Hypothermia and cell survival pathways

Interestingly, while TH inhibits many molecules associated with apoptotic cell death, it can also upregulate several factors involved in cell survival. Part of the reason may be due to the upregulation of cold stress proteins, which are induced at lower body temperatures. RNA-binding motif protein 3 (RBM3), perhaps the best studied of these cold stress proteins, has been shown to increase under hypothermic conditions after stroke in laboratory and clinical studies (54) and is associated with neuroprotection (55). TH has also been shown to increase the expression of brain-derived neurotrophic factor (BDNF), glial-derived neurotrophic factor (GNF), and neurotrophin (NT) in ischemia-injured brain (56–58). Mild hypothermia induced phosphorylation of extracellular signal-regulated kinase-1/2 (ERK1/2), which is downstream of BDNF (56), and has also been shown to activate Akt, which is a serine/threonine protein kinase that has multiple roles in cellular plasticity and migration (59). Downstream of Akt, phosphatase and tensin homolog (PTEN) may lead to apoptosis, but in its phosphorylated state, it promotes cell survival. Phosphorylated PTEN is also known to prevent neuronal cell death in ischemic brain injury (53), and TH preserves phosphorylated PTEN levels in stroke models (60). These observations indicate that while brain cooling suppresses metabolic and cellular functions, lower temperatures also permit the upregulation of other factors that ultimately promote cell survival.

### Anti-inflammatory effects of hypothermia

Following acute ischemic stroke, necrotic cells, cell debris, and increased reactive oxygen species (ROS) induce inflammatory responses, which are related to activating microglia and infiltrating leukocytes from circulating blood to parenchymal brain tissue. The recruitment of both brain and peripheral immune cells in post-ischemic tissue can accelerate and expand an infarct initiated by ischemia (61). Mild hypothermia suppresses microglial activation and tissue viability after ischemic stroke (35) and prevents infiltrating neutrophils from entering ischemic brain tissue. TH has also been shown to reduce levels of many inflammatory mediators, such as adhesion molecules, ROS, and pro-inflammatory cytokines (62–64).

TH also affects the activity of transcription factors involved in inflammatory responses, such as nuclear factor-κB (NF-κB) (65). In brain ischemia models, TH has been shown to prevent NF-κB translocation to the nucleus and preventing the upregulation of target pro-inflammatory genes (66). TH has similarly been shown to inhibit other transcription factors, including mitogen-activated protein kinase (MAPK), and Janus kinases/signal transducer and activator of transcription proteins (JAK/STATs), all of which have been documented to elicit inflammation following stroke (67, 68).

Cytokines, which play an important role in immune system signaling and potentiation of immune responses, are quickly and extensively upregulated in brain tissue after ischemic stroke (69). TH has been shown to suppress many inflammatory cytokines, including transforming growth factor (TGF)-β, tumor necrosis factor-α (TNF-α), and several interleukins (IL) (59, 64). TH has also been shown to induce an anti-inflammatory M2 phenotype in microglia in stroke models (70). Recent work has shown that even a low normal temperature of 36°C can attenuate protein levels of several pro-inflammatory cytokines (71), and temperature modulation to both 33 and 36°C can provide equivalent neuroprotection by decreasing pro-inflammatory M1 activation of microglia and by relatively increasing microglial polarization toward a beneficial M2 phenotype (71). Considering recent concerns at the clinical level that TH to temperatures of 32–34°C may be poorly tolerated in some patients, it is indicated that a body temperature of 36°C may be better tolerated clinically and may have some therapeutic and anti-inflammatory effects (71).

### Differential protection within the neurovascular unit

While neuroprotection in laboratory studies has shown fairly consistent and robust results in various disease models, the same cannot be said at the clinical level. Thus, taking into consideration various cells that comprise the neurovascular unit, Lyden et al. (72) systematically examined the protective effects of cooling in cultures of neurons, astrocytes, and brain-derived endothelial cells and found considerable differences in their response to cooling. In particular, they found that cooling inhibited astrocyte activation and seemed to prevent any beneficial effect of paracrine factors that astrocytes provide neurons. The study also found that different cell populations, while showing a similar pattern of dose-dependent responses to the depth and duration of cooling and finite responses to cooling delays, the time frame was different depending on the cell type. In line with prior reports (73), the investigators found that lower temperatures offered more robust protection, and that delaying the onset of cooling required longer cooling times. Yet, they also warned that longer cooling times could also interrupt the paracrine functions of astrocytes, which, in turn, could reduce the effectiveness of the therapy. Subsequent studies by other laboratories have since shown that cooling improves other properties of astrocytes, such as their ability to transfer viable mitochondria to injured neurons (74) and that their phenotype can shift from an activated (A1) to a non-activated (A2) state (75). Thus, these recent laboratory observations should be considered in the design of future clinical trials.

## Clinical application and adverse effects of systemic therapeutic hypothermia

The clinical application of TH has become more effective, simpler, and safer by the development of automated cooling devices through intravascular or surface cooling (16, 76, 77). In practice, TH consists of three critical phases: induction, maintenance, and rewarming (16, 78). Induction involves minimizing ongoing irreversible neuronal injury due to ischemia and reperfusion, and target temperature should be reached as rapidly as possible, typically by using ice packs and infusion of cold intravenous fluids (77). During the maintenance phase after the target temperature is reached, core temperature should be maintained by using advanced cooling technologies (± 0.5°C of the target temperature) (79). One of the many benefits of TH during the maintenance phase is decreased intracranial pressure (ICP). The most robust clinical benefit of TH on ICP is when systemic body temperature decreases to <35.5°C (16, 17, 80). The rewarming phase can be the most detrimental phase in TH if body temperature is increased too rapidly. This can lead to systemic vasodilation and hypotension. As a consequence, rapid rewarming can induce cerebral vasodilation and lead to abrupt ICP increases (21, 81, 82), which can lead to bad outcomes, especially in patients with brain edema (83).

In clinical trials, long durations of TH have been limited by feasibility and several complications of cooling (84). Preclinical studies revealed that transient and rebound elevation of ICP may occur during rewarming 18–24 h after stroke, which leads to neurological deterioration (84–86). Short (2.5 h) and ultra-short durations (30 min) of TH could prevent significant ICP elevations (84, 85), although cooling to 34.5°C with ultra-short durations did not significantly prevent ICP elevation and did not decrease infarct volume (84). As such, the authors suggested that milder hypothermia (≥ 34.5°C) requires a longer duration at target temperature (84). It remains to be seen whether short-duration mild hypothermia can provide the same benefits of TH in clinical practice. Although further preclinical studies need to validate the therapeutic benefits of short-duration cooling in patients with stroke, short- and ultra-short-duration cooling could be an alternative to minimize complications and maximize therapeutic effects.

Hypothermia also induces shivering in awake individuals as body temperature reaches about 35.5°C in healthy individuals. It is a major adverse effect as it interferes with the cooling process by making it difficult to reach the target temperature and causes detrimental effects through its massive increase in metabolic demand and energy consumption both systemically and by the brain (16, 76, 87). If shivering can be controlled, TH is then able to decrease systemic and cerebral metabolism and permit the beneficial effects of cooling to proceed. Lowered temperatures are not without risk. In clinical studies, TH has been shown to reduce electrolyte levels, such as that of potassium, phosphate, and magnesium (88). In addition, carbon dioxide partial pressure (pCO2) is lower and blood pH is increased because carbon dioxide becomes more soluble in hypothermic patients (89). This phenomenon called alkaline shift can occur during cooling. Hypothermia can also lead to hyperglycemia due to insulin resistance, while rewarming after hypothermia can induce hypoglycemia through a rapid increase in insulin sensitivity (90). Hypothermia, even mild hypothermia, is capable of inducing cardiac arrhythmias such as atrial and ventricular tachycardia and fibrillation. In general, these arrhythmias can be precipitated when core temperatures fall below 32°C, but those with underlying cardiac conditions may be more sensitive to cooling and develop arrhythmias at higher temperatures. In most cases, the heart tolerates hypothermia in temperature ranges between 32°C and 35°C (90, 91). As hypothermia impairs immune function, coagulation, and platelet function, cooling can increase the risk of infections, such as pneumonia, coagulopathy, and bleeding (16, 92).

## Current state of systemic therapeutic hypothermia for acute ischemic stroke

At the clinical level, current practices suggest lowering body and/or brain temperature for 12–24 h to 32–34°C. This is based on both experimental studies and clinical studies in comatose survivors of cardiac arrest (14). Although it is well documented that therapeutic hypothermia has beneficial effects in many animal models of ischemic and traumatic brain injury, further larger clinical trials are needed to validate its therapeutic efficacy and its full translation in humans in the treatment of acute ischemic stroke (73, 93, 94). Currently, there are no large phase III studies to evaluate TTM for acute ischemic stroke. The Cooling for Acute Ischemic Brain Damage (COOL-AID) trial was a small trial conducted to assess feasibility of moderate cooling of 32–33°C using an endovascular device in patients with acute ischemic stroke (95, 96). There was no significant improvement in modified Rankin scores (mRS) and mortality in the hypothermic group in comparison with the control group, although the trial was not intended to study efficacy (95, 96).

The Intravascular Cooling Trial of Acute Stroke (ICTuS) demonstrated safety of 12–24 h of intravascular cooling in patients with stroke (23, 25). It also tested a protocol to reduce shivering in normally awake patients with stroke and found that an anti-shivering regimen with buspirone and meperidine could be given in this patient population. A subsequent trial, the Intravascular Cooling in the Treatment of Stroke-Longer tissue plasminogen activator (tPA) Window (ICTuS-L) trial, also showed acceptable safety and feasibility for intravascular TH in patients who received intravenous tPA (24, 25). Unfortunately, there was no significant improvement in modified Rankin scores (mRS) and mortality in the hypothermic group compared with the control group (24). They also reported significantly increased risks of pneumonia in the ICTuS-L patients who received TH (25, 97). To provide safer, faster TH in patients with stroke, an expanded trial, ICTuS-2, failed to reduce the risk of pneumonia and mortality in the TH-treated group (25). The COOL-AID and the ICTuS-L trials highlighted significant clinical issues associated with TH, such as shivering control, airway management, and prevention of complications (98–100).

In patients with malignant middle cerebral artery (MCA) stroke, TH to 33°C ± 1°C after early decompressive hemicraniectomy had no significant improvement in mortality at 14 days and neurological outcomes at 12 months in comparison to the control group that does not allow actively lowering beneath 36.5°C (101, 102). Unfortunately, the study showed that the TH group after early decompressive hemicraniectomy had a higher rate of serious adverse effects than the control group (102). To identify the benefits of hypothermia on the clinical outcomes of patients with large hemispheric infarction (LHI), Li et al. performed systematic review and meta-analysis (103) and found that there was no significant benefit between application of hypothermia and mortality. They also found that there was a higher risk of several adverse effects. Yet, TH in patients with LHI had improved neurological outcomes compared with patients with LHI who did not receive cooling (103). Future studies are also necessary to demonstrate the safety and effectiveness of hypothermia for LHI.

Despite several clinical trials in ischemic stroke, none have proved the effectiveness of TTM in this patient population (104). Although some studies showed a positive trend toward better outcomes, a systemic review and meta-analysis by Kuczynski et al. (105) did not find that TTM after acute ischemic stroke provided beneficial effects. Unfortunately, TH increased risks of complications in patients with stroke who are typically older with more medical comorbidities than other patient populations (93, 105), although one study by Hong et al. (106) showed lower rates of pneumonia in patients who received TH after stroke than reported in other studies. In their study, all patients receiving TH underwent mechanical ventilation; this may represent a critical difference in the therapeutic protocols for hypothermia in cardiac arrest studies compared with stroke studies and suggest the importance of a secure airway for the prevention of pneumonia (106). Furthermore, most patients with stroke remain awake and breathe spontaneously during the acute phase of stroke (16), compared with studies of TTM in comatose survivors of cardiac arrest. Because of older age and comorbidities in these patients, it may be dangerous to employ sedative and anti-shivering agents, advanced airway management, and mechanical ventilation. Sedation and respiratory depression that may result from the use of pharmacological anti-shivering agents can impede the neurological examination and aggravate hypoxia and hypercarbia. These are additional reasons that any neuroprotective effects of TH may not be observed in patients with stroke (16). Since systemic hypothermia can affect all organ systems in the human body, the use of TTM should be based on the balance between its potential benefits and harm (18).

In spite of many setbacks at the clinical level, TTM still has the strongest evidence for neuroprotection in preclinical studies. There is still much to be learned about the underlying mechanisms of this protection, and aspects of hypothermic neuroprotection could be leveraged to develop an effective yet safe therapy for patients with stroke.

## Clinical challenges of therapeutic hypothermia for acute ischemic stroke

### Selective brain cooling

Since whole body cooling to achieve lower brain temperatures can inadvertently lead to systemic complications, selective brain cooling has the potential to avoid these complications while potentially offering robust neuroprotection to the brain. Selective brain cooling lowers temperature of the brain to below the core temperature by inducing a net temperature gradient between the brain and the rest of the body (107). This approach has been shown to be feasible and resulted in the same benefits of TH in a variety of animal models. Particularly for patients with stroke, this approach is an attractive alternative to systemic hypothermia for neuroprotection (108). In general, selective brain cooling promotes more rapid, profound, organ-specific induction and maintenance of target temperature of the brain in comparison with systemic hypothermia (107, 108).

Selective brain cooling consists of both non-invasive and invasive cooling methods (109). Non-invasive approaches include local surface cooling such as a cooling helmet, cap, or a head-and-neck cooling device that could induce a median gradient of temperature between the brain and the core of 3.4°C. Selective brain cooling was shown to be feasible in newborn pigs subjected to a global ischemic insult (110). Selective brain cooling in clinical studies of severe traumatic brain injury also led to improved neurological outcomes without severe complications in patients receiving cooling caps and neckbands after severe traumatic brain injury (107, 110, 111). Another approach is that of intranasal cooling, which leads to heat exchange by direct heat loss to air and evaporation of water, which can reach up to a 10% rate of total body heat loss in normal conditions (107). Transnasal evaporative cooling has been shown to improve neurological outcomes in patients who received cardiopulmonary resuscitation after cardiac arrest with acceptable feasibility and safety (112, 113). However, non-invasive cooling using surface cooling and nasopharyngeal cooling is relatively less effective in lowering temperature of the brain parenchyma (20). Furthermore, since surface cooling will ultimately lead to local cooling of circulating blood, selective surface cooling has the potential to cool other parts of the body as well.

Invasive selective cooling includes epidural, subdural, and subarachnoid cooling; retrograde jugular venous cooling; and intraarterial selective cooling (20). Despite the effectiveness of invasive selective cooling, these methods lead to increased risks of infection and intracranial hemorrhage, thus limiting the application of these approaches in clinical settings (107).

Nevertheless, selective brain cooling has been increasingly embraced by those involved in the care of patients with acute ischemic stroke due to the challenges of whole body cooling outlined earlier (20). Since endovascular embolectomy has been shown to have a role in the treatment of acute ischemic stroke in recent years, selective endovascular infusion of cold saline directly into the brain parenchyma offers a promising strategy to induce more rapid target temperature decreases to the brain through preexisting angiographic catheters (20, 114). In animal models, local endovascular infusion of 23°C saline at 2 mL/min for 3–4 min before reperfusion leads to lowering of brain temperature within 10 min, and local endovascular infusion (10°C saline at 0.25 mL/min) after reperfusion leads to decreased brain temperature to <35°C by 20 min (115). In the Copenhagen Stroke Study, whole body surface cooling was able to reduce body temperature by a mean of 1.3°C without the use of anesthesia in awake patients with stroke (116, 117). However, a significantly lower mean body temperature was achieved after 1 and 6 h of hypothermic therapy [t_0_ = 36.8°C vs. t_1_ = 36.4, *P* = 0.002 and (t_0_ = 36.8°C vs. t_6_ = 35.5°C, *P* < 0.001)] (116). Since the time window for therapeutic intervention after ischemic insults is thought to be a matter of minutes to hours, rapid recognition of injury and rapid institution of treatments are highly desirable in patients with acute ischemic stroke (115). Using selective endovascular infusion, target temperature can be reached more quickly than the classical surface cooling and systemic infusion of cold saline (115). Initiating selective cooling before reperfusion significantly reduces the infarct volume by 75–90% and preserves motor function after ischemic stroke (114, 118, 119). Several studies reported that postreperfusion selective hypothermia improved infarct volume and neurological recovery in animal models of stroke (120, 121). Another neuroprotective benefit of local selective endovascular infusion over systemic infusion is washout of by-products of metabolism induced by ischemia, which can lead to vasodilatation (122). This washout can minimize the extent of reperfusion injury that may be elicited by hyperperfusion (115). Selective cooling through local endovascular infusion was recently verified to have acceptable safety and feasibility profiles not only in animals but also in humans (115).

In humans, Choi et al. studied 18 patients undergoing cerebral angiography who received infusion of isotonic saline (4–17°C) at 33 mL/min for 10 min into an internal carotid artery (123). Cold saline infusion led to a 0.84°C decrease in jugular venous blood temperature (123), while systemic temperature was changed by 0.15°C from baseline (123). These investigators also did not report any serious complications, such as vasospasm, microemboli, pain, and focal neurologic deficits (123).

In a few small, non-randomized, observational studies, Chen et al. demonstrated that selective brain cooling by at least 2°C in brain tissue through intraarterial infusion of 4°C cold saline combined with endovascular recanalization was feasible and safe in patients with acute ischemic stroke (122, 124). A prospective non-randomized cohort study of 113 consecutive patients with acute ischemic stroke reported that infarct volume was reduced at 3–7 days with acceptable safety in a cohort that received mechanical thrombectomy combined with intraarterial selective cooling infusion. However, at 90 days, no differences were found in the proportion of patients who achieved functional independence (mRS 0–2) (20, 109). To date, the clinical effectiveness of selective endovascular brain cooling remains to be determined in ischemic stroke patients with large-vessel occlusions to the brain (125). However, vessel recanalization by thrombolytic and mechanical recanalization therapies has been shown to improve functional outcomes in acute stroke and selective endovascular cooling could be fairly easily incorporated into clinical practice (2, 6, 126). Selective brain cooling combined with recanalization can also be a more effective strategy since preclinical studies have shown that TH is more effective when reperfusion occurs (115). Recently, novel selective cooling systems have been developed as a combination of nasopharyngeal brain and endovascular cooling systems (127). As recent technological advances, the application of sensors, artificial intelligence, and smart devices have the potential to reduce complications and maximize the therapeutic effect of selective brain cooling. Further studies are needed to explore optimal protocols for selective brain cooling that lead to improved clinical outcomes in patients with stroke.

### Targeted temperature management at or near normothermia

TTM encompasses therapeutic hypothermia, mild therapeutic hypothermia, controlled normothermia, and treatment of fever (128). Subsequent work in the area of TTM for comatose survivors of cardiac arrest has explored the feasibility and therapeutic value of reducing the target temperature to 36°C (18). In 2013, a randomized controlled trial compared mortality and neurologic outcomes after the application of TTM in patients with cardiac arrest. Body temperatures of 33°C and 36°C were compared, and the benefits of TTM at 36°C were not inferior to those of TTM at 33°C (18). Furthermore, managing shivering and rewarming also maximizes the chances of maximal beneficial effects of TH (19).

Based on these studies and those conducted before, the selection and maintenance of target temperatures between 32°C and 36°C for at least 24 h were proposed as optimal for TTM by the 2015 Cardiopulmonary Resuscitation and Emergency Cardiovascular Care guidelines (92). TTM at 36°C has several advantages over lower temperatures as it may be easier to prevent shivering in the induction phase, reduce the risk of intracranial pressure increases in the rewarming phase, provide a safer margin for serious hypothermia-induced cardiac arrhythmias, and diminish the risk of other systemic detrimental effects of lower temperatures, such as renal dysfunction, coagulopathy, pneumonia, and sepsis. As the effects of TTM at 36°C were no different from 33°C after cardiac arrest, TTM at 36°C may prove more attractive in the treatment of patients with stroke. However, it should be noted that the aforementioned study did not include a control group, where body temperature was maintained in the normal range for comparison.

Nevertheless, the damaging effects of hyperthermia and fever are well known in stroke (117, 129). In patients surviving cardiac arrest, the development of fever is also known to increase the risk for poor neurologic outcomes (130). Accordingly, the randomized Targeted Hypothermia vs. Targeted Normothermia after Out-of-Hospital Cardiac Arrest (TTM2) trial was conducted to identify the beneficial and harmful effects of hypothermia in comparison with normothermia and early control of fever. As the TTM2 trial was limited to patients with out-of-hospital cardiac arrest (OHCA) of a presumed cardiac or unknown cause, this trial result is not universally accepted for stroke and other presentations of cardiac arrest (131, 132). In addition, there was no group without TTM; thus, this trial did not propose a therapeutic benefit between any active control of core temperature and no control (132). In common practice, TTM applications could be limited (131, 132). Future trials are necessary to answer questions raised by TTM2.

Patients treated with TTM after OHCA did not have reduced mortality at 6 months compared with those with controlled normothermia (132). Aneurysmal subarachnoid hemorrhage guidelines now recommend aggressive control of fever to near normothermia to prevent delayed cerebral ischemia due to vasospasm (133). Piironen et al. (134) studied a cohort of patients with stroke and compared neurological outcomes between those treated with mild hypothermia with 35°C with a surface cooling device and standard stroke unit care with the core temperatures maintained at <37.5°C within 6 h of symptom onset. No differences were found in favorable outcomes between the groups at 3 months (134). The rate of adverse events was lower in patients who received mild hypothermia than in those who received moderate hypothermia in previous studies (134). The mortality rate in their study among the hypothermia group was 0% in comparison to 28% in the COOL AID II, 21% in the ICTuS-L, and 12% in the COOLAID Oresund trial (129, 134). Despite the adverse events and small doses of sedatives and anti-shivering medications, mild hypothermia to 35°C with a surface cooling device appears safe and feasible in spontaneously breathing and awake patients with stroke who received intravenous thrombolysis (129, 134). From a different perspective, controlled normothermia or TTM near normothermia may lead to similar neurologic outcomes without the requirement of sedative or anti-shivering agents, and with more severe adverse effects. However, further prospective multicenter studies are needed to validate the potential benefits of TTM to controlled or near normothermia.

## Perspectives

Therapeutic hypothermia may be the most robust neuroprotective modality in laboratory and preclinical studies. Neuroprotective effects have been associated with beneficial effects on metabolic, molecular, and inflammation-related events in cell death; however, clinical studies in patients with stroke have yet to demonstrate a clear benefit compared with favorable outcomes shown in other populations such as comatose survivors of cardiac arrest and neonatal ischemic encephalopathy. Recent laboratory observations shed light on differential vulnerabilities to cells that comprise the neurovascular unit that could drive more careful study of how to best apply TTM depending on factors such as the delay in cooling, which could drive the depth and duration of TTM therapy. To improve clinical outcomes by TTM in patients with stroke, numerous studies have collectively contributed important knowledge to overcome clinical obstacles. For TTM to be effective, faster induction, minimal impact on core temperature, and ameliorating systemic adverse effects have been significant challenges. Shivering has been perhaps the most challenging obstacle when using the surface cooling device in awake patients. Airway management has also been shown to be an important factor in good outcomes in patients with stroke treated with TTM to reduce complications, such as aspiration and pneumonia. Safety and feasibility studies of TH have revealed the challenges of TTM in patients with stroke, particularly those of older age and those with multiple comorbidities. It may also be necessary to apply both systemic and selective cooling modalities for optimal TH. Further preclinical and clinical studies are still needed to assess and optimize the indications, target core temperatures, rate, and depth of cooling as well as method, initiation, maintenance, and rewarming for cooling protocols. The potential for normothermia and near normothermia TTM should be further explored in this patient population as well. Much progress has been made for therapeutic cooling and temperature management in patients with stroke, but there is still more work to be undertaken.

## Author contributions

All authors listed have made a substantial, direct, and intellectual contribution to the work and approved it for publication.

## Funding

This study was supported by grants from the National Research Foundation of Korea (NRF) funded by the Ministry of Education (NRF-2020R1I1A1A01064803 to JK) and by the Ministry of Science, ICT and Future Planning (NRF- 2021R1C1C1009209 to JY); a faculty research grant from Yonsei University College of Medicine for 2019 (6-2019-0188); and NIH NINDS R01 NS106441 (MY). The grant to MY was administered by the Northern California Institute for Research and Education and supported by resources of the Veterans Affairs Medical Center, San Francisco, California.

## Conflict of interest

The authors declare that the research was conducted in the absence of any commercial or financial relationships that could be construed as a potential conflict of interest.

## Publisher's note

All claims expressed in this article are solely those of the authors and do not necessarily represent those of their affiliated organizations, or those of the publisher, the editors and the reviewers. Any product that may be evaluated in this article, or claim that may be made by its manufacturer, is not guaranteed or endorsed by the publisher.

## References

[1] Virani SS Alonso A Benjamin EJ Bittencourt MS Callaway CW Carson AP. Heart disease and stroke statistics-2020 update: a report from the American Heart Association. Circulation. (2020) 141:e139–596. 10.1161/CIR.0000000000000746 31992061

[2] Hankey GJ. Stroke. Lancet. (2017) 389:641–54. 10.1016/S0140-6736(16)30962-X 27637676

[3] Fonarow GC Zhao X Smith EE Saver JL Reeves MJ Bhatt DL. Door-to-needle times for tissue plasminogen activator administration and clinical outcomes in acute ischemic stroke before and after a quality improvement initiative. JAMA. (2014) 311:1632–40. 10.1001/jama.2014.3203 24756513

[4] Chen ZM Sandercock P Pan HC Counsell C Collins R Liu LS. Indications for early aspirin use in acute ischemic stroke: a combined analysis of 40 000 randomized patients from the chinese acute stroke trial and the international stroke trial. On behalf of the CAST and IST collaborative groups. Stroke. (2000) 31:1240–9. 10.1161/01.STR.31.6.1240 10835439

[5] Smith WS Sung G Saver J Budzik R Duckwiler G Liebeskind DS. Mechanical thrombectomy for acute ischemic stroke: final results of the Multi MERCI trial. Stroke. (2008) 39:1205–12. 10.1161/STROKEAHA.107.497115 18309168

[6] Powers WJ. Acute ischemic stroke. N Engl J Med. (2020) 383:252–60. 10.1056/NEJMcp1917030 32668115

[7] Prabhakaran S Ruff I Bernstein RA. Acute stroke intervention: a systematic review. JAMA. (2015) 313:1451–62. 10.1001/jama.2015.3058 25871671

[8] Lacy CR Suh DC Bueno M Kostis JB. Delay in presentation and evaluation for acute stroke: stroke time registry for outcomes knowledge and epidemiology (S.T.R.O.K.E.). Stroke. (2001) 32:63–9. 10.1161/01.STR.32.1.63 11136916

[9] Nedeltchev K Arnold M Brekenfeld C Isenegger J Remonda L Schroth G. Pre- and in-hospital delays from stroke onset to intra-arterial thrombolysis. Stroke. (2003) 34:1230–4. 10.1161/01.STR.0000069164.91268.99 12702836

[10] Kharbach A Obtel M Achbani A Aasfara J Hassouni K Lahlou L. Ischemic stroke in Morocco: Prehospital delay and associated factors. Rev Epidemiol Sante Publique. (2021) 69:345–59. 10.1016/j.respe.2021.03.010 34148762

[11] Evenson KR Foraker RE Morris DL Rosamond WD. A comprehensive review of prehospital and in-hospital delay times in acute stroke care. Int J Stroke. (2009) 4:187–99. 10.1111/j.1747-4949.2009.00276.x 19659821 PMC2825147

[12] Donnan GA Davis SM Parsons MW Ma H Dewey HM Howells DW. How to make better use of thrombolytic therapy in acute ischemic stroke. Nat Rev Neurol. (2011) 7:400–9. 10.1038/nrneurol.2011.89 21670766

[13] Yenari MA Hemmen TM. Therapeutic hypothermia for brain ischemia: where have we come and where do we go? Stroke. (2010) 41:S72–4. 10.1161/STROKEAHA.110.595371 20876510 PMC2953728

[14] Bernard SA Gray TW Buist MD Jones BM Silvester W Gutteridge G. Treatment of comatose survivors of out-of-hospital cardiac arrest with induced hypothermia. N Engl J Med. (2002) 346:557–63. 10.1056/NEJMoa003289 11856794

[15] Mild therapeutic hypothermia to improve the neurologic outcome after cardiac arrest. N Engl J Med. (2002) 346:549–56. 10.1056/NEJMoa012689 11856793

[16] Choi HA Badjatia N Mayer SA. Hypothermia for acute brain injury–mechanisms and practical aspects. Nat Rev Neurol. (2012) 8:214–22. 10.1038/nrneurol.2012.21 22371279

[17] Edwards AD Brocklehurst P Gunn AJ Halliday H Juszczak E Levene M. Neurological outcomes at 18 months of age after moderate hypothermia for perinatal hypoxic ischaemic encephalopathy: synthesis and meta-analysis of trial data. BMJ. (2010) 340:c363. 10.1136/bmj.c363 20144981 PMC2819259

[18] Nielsen N Wetterslev J Cronberg T Erlinge D Gasche Y Hassager C. Targeted temperature management at 33°C versus 36°C after cardiac arrest. N Engl J Med. (2013) 369:2197–206. 10.1056/NEJMoa1310519 24237006

[19] Lee JH Lim J Chung YE Chung SP Park I Kim CH. Targeted temperature management at 33°C or 36°C produces equivalent neuroprotective effects in the middle cerebral artery occlusion rat model of ischemic stroke. Shock. (2018) 50:714–9. 10.1097/SHK.0000000000001106 29337840

[20] Wu L Wu D Yang T Xu J Chen J Wang L. Hypothermic neuroprotection against acute ischemic stroke: the 2019 update. J Cereb Blood Flow Metab. (2020) 40:461–81. 10.1177/0271678X19894869 31856639 PMC7026854

[21] Clifton GL Valadka A Zygun D Coffey CS Drever P Fourwinds S. Very early hypothermia induction in patients with severe brain injury (the National Acute Brain Injury Study: Hypothermia II): a randomised trial. Lancet Neurol. (2011) 10:131–9. 10.1016/S1474-4422(10)70300-8 21169065 PMC3628679

[22] Todd MM Hindman BJ Clarke WR Torner JC. Mild intraoperative hypothermia during surgery for intracranial aneurysm. N Engl J Med. (2005) 352:135–45. 10.1056/NEJMoa040975 15647576

[23] Lyden PD Allgren RL Ng K Akins P Meyer B Al-Sanani F. Intravascular Cooling in the Treatment of Stroke (ICTuS): early clinical experience. J Stroke Cerebrovasc Dis. (2005) 14:107–14. 10.1016/j.jstrokecerebrovasdis.2005.01.001 17904009

[24] Hemmen TM Raman R Guluma KZ Meyer BC Gomes JA Cruz-Flores S. Intravenous thrombolysis plus hypothermia for acute treatment of ischemic stroke (ICTuS-L): final results. Stroke. (2010) 41:2265–70. 10.1161/STROKEAHA.110.592295 20724711 PMC2947593

[25] Lyden P Hemmen T Grotta J Rapp K Ernstrom K Rzesiewicz T. Results of the ICTuS 2 Trial (Intravascular Cooling in the Treatment of Stroke 2). Stroke. (2016) 47:2888–95. 10.1161/STROKEAHA.116.014200 27834742 PMC5134910

[26] Yenari MA Han HS. Neuroprotective mechanisms of hypothermia in brain ischaemia. Nat Rev Neurosci. (2012) 13:267–78. 10.1038/nrn3174 22353781

[27] Poli S Purrucker J Priglinger M Diedler J Sykora M Popp E. Induction of cooling with a passive head and neck cooling device: effects on brain temperature after stroke. Stroke. (2013) 44:708–13. 10.1161/STROKEAHA.112.672923 23339959

[28] Gluckman PD Wyatt JS Azzopardi D Ballard R Edwards AD Ferriero DM. Selective head cooling with mild systemic hypothermia after neonatal encephalopathy: multicentre randomised trial. Lancet. (2005) 365:663–70. 10.1016/S0140-6736(05)70932-6 15721471

[29] Wang H Olivero W Lanzino G Elkins W Rose J Honings D. Rapid and selective cerebral hypothermia achieved using a cooling helmet. J Neurosurg. (2004) 100:272–7. 10.3171/jns.2004.100.2.0272 15086235

[30] Shackelford RT Hegedus SA. Factors affecting cerebral blood flow–experimental review: sympathectomy, hypothermia, CO2 inhalation and pavarine. Ann Surg. (1966) 163:771–7. 10.1097/00000658-196605000-00014 5930460 PMC1477179

[31] Hägerdal M Harp J Nilsson L Siesjö BK. The effect of induced hypothermia upon oxygen consumption in the rat brain. J Neurochem. (1975) 24:311–6. 10.1111/j.1471-4159.1975.tb11881.x 1113108

[32] Ito U Hakamata Y Kawakami E Oyanagi K. Temporary [corrected] cerebral ischemia results in swollen astrocytic end-feet that compress microvessels and lead to delayed [corrected] focal cortical infarction. J Cereb Blood Flow Metab. (2011) 31:328–38. 10.1038/jcbfm.2010.97 20588315 PMC3049496

[33] Kurisu K Abumiya T Nakamura H Shimbo D Shichinohe H Nakayama N. Transarterial regional brain hypothermia inhibits acute aquaporin-4 surge and sequential microvascular events in ischemia/reperfusion injury. Neurosurgery. (2016) 79:125–34. 10.1227/NEU.0000000000001088 26516820

[34] Lee JM Zipfel GJ Choi DW. The changing landscape of ischaemic brain injury mechanisms. Nature. (1999) 399:A7–14. 10.1038/399a007 10392575

[35] Van Hemelrijck A Vermijlen D Hachimi-Idrissi S Sarre S Ebinger G Michotte Y. Effect of resuscitative mild hypothermia on glutamate and dopamine release, apoptosis and ischaemic brain damage in the endothelin-1 rat model for focal cerebral ischaemia. J Neurochem. (2003) 87:66–75. 10.1046/j.1471-4159.2003.01977.x 12969253

[36] Kim JY Kim N Yenari MA Chang W. Mild Hypothermia Suppresses Calcium-Sensing Receptor (CaSR) induction following forebrain ischemia while increasing GABA-B receptor 1 (GABA-B-R1) expression. Transl Stroke Res. (2011) 2:195–201. 10.1007/s12975-011-0082-4 21731589 PMC3124781

[37] Lyden PD Krieger D Yenari M Dietrich WD. Therapeutic hypothermia for acute stroke. Int J Stroke. (2006) 1:9–19. 10.1111/j.1747-4949.2005.00011.x 18706063

[38] Xu L Yenari MA Steinberg GK Giffard RG. Mild hypothermia reduces apoptosis of mouse neurons in vitro early in the cascade. J Cereb Blood Flow Metab. (2002) 22:21–8. 10.1097/00004647-200201000-00003 11807390

[39] Shimohata T Zhao H Steinberg GK Epsilon PKC. May contribute to the protective effect of hypothermia in a rat focal cerebral ischemia model. Stroke. (2007) 38:375–80. 10.1161/01.STR.0000254616.78387.ee 17204679

[40] Kim JY Kim N Lee JE Yenari MA. Hypothermia identifies dynamin as a potential therapeutic target in experimental stroke. Ther Hypothermia Temp Manag. (2017) 7:171–7. 10.1089/ther.2017.0005 28665255 PMC5610406

[41] Green DR Reed JC. Mitochondria and apoptosis. Science. (1998) 281:1309–12. 10.1126/science.281.5381.1309 9721092

[42] Liu L Yenari MA. Therapeutic hypothermia: neuroprotective mechanisms. Front Biosci. (2007) 12:816–25. 10.2741/2104 17127332

[43] Prakasa Babu P Yoshida Y Su M Segura M Kawamura S Yasui N. Immunohistochemical expression of Bcl-2, Bax and cytochrome c following focal cerebral ischemia and effect of hypothermia in rat. Neurosci Lett. (2000) 291:196–200. 10.1016/S0304-3940(00)01404-X 10984640

[44] Slikker W. 3rd, Desai VG, Duhart H, Feuers R, Imam SZ Hypothermia enhances bcl-2 expression and protects against oxidative stress-induced cell death in Chinese hamster ovary cells. Free Radic Biol Med. (2001) 31:405–11. 10.1016/S0891-5849(01)00593-7 11461779

[45] Zhang Z Sobel RA Cheng D Steinberg GK Yenari MA. Mild hypothermia increases Bcl-2 protein expression following global cerebral ischemia. Brain Res Mol Brain Res. (2001) 95:75–85. 10.1016/S0169-328X(01)00247-9 11687278

[46] Inamasu J Suga S Sato S Horiguchi T Akaji K Mayanagi K. Postischemic hypothermia attenuates apoptotic cell death in transient focal ischemia in rats. Acta Neurochir Suppl. (2000) 76:525–7. 10.1007/978-3-7091-6346-7_110 11450083

[47] Ferrer I Planas AM. Signaling of cell death and cell survival following focal cerebral ischemia: life and death struggle in the penumbra. J Neuropathol Exp Neurol. (2003) 62:329–39. 10.1093/jnen/62.4.329 12722825

[48] Liu L Kim JY Koike MA Yoon YJ Tang XN Ma H. FasL shedding is reduced by hypothermia in experimental stroke. J Neurochem. (2008) 106:541–50. 10.1111/j.1471-4159.2008.05411.x 18410517 PMC2735469

[49] Lee JE Yoon YJ Moseley ME Yenari MA. Reduction in levels of matrix metalloproteinases and increased expression of tissue inhibitor of metalloproteinase-2 in response to mild hypothermia therapy in experimental stroke. J Neurosurg. (2005) 103:289–97. 10.3171/jns.2005.103.2.0289 16175859

[50] Truettner JS Alonso OF Dietrich WD. Influence of therapeutic hypothermia on matrix metalloproteinase activity after traumatic brain injury in rats. J Cereb Blood Flow Metab. (2005) 25:1505–16. 10.1038/sj.jcbfm.9600150 15959464

[51] Broughton BR Reutens DC Sobey CG. Apoptotic mechanisms after cerebral ischemia. Stroke. (2009) 40:e331–9. 10.1161/STROKEAHA.108.531632 19182083

[52] Susin SA Lorenzo HK Zamzami N Marzo I Snow BE Brothers GM. Molecular characterization of mitochondrial apoptosis-inducing factor. Nature. (1999) 397:441–6. 10.1038/17135 9989411

[53] Zhao H Wang JQ Shimohata T Sun G Yenari MA Sapolsky RM. Conditions of protection by hypothermia and effects on apoptotic pathways in a rat model of permanent middle cerebral artery occlusion. J Neurosurg. (2007) 107:636–41. 10.3171/JNS-07/09/0636 17886565

[54] Ávila-Gómez P Vieites-Prado A Dopico-López A Bashir S Fernández-Susavila H Gubern C. Cold stress protein RBM3 responds to hypothermia and is associated with good stroke outcome. Brain Commun. (2020) 2:fcaa078. 10.1093/braincomms/fcaa078 33585816 PMC7869850

[55] Chip S Zelmer A Ogunshola OO Felderhoff-Mueser U Nitsch C Bührer C. The RNA-binding protein RBM3 is involved in hypothermia induced neuroprotection. Neurobiol Dis. (2011) 43:388–96. 10.1016/j.nbd.2011.04.010 21527344

[56] Vosler PS Logue ES Repine MJ Callaway CW. Delayed hypothermia preferentially increases expression of brain-derived neurotrophic factor exon III in rat hippocampus after asphyxial cardiac arrest. Brain Res Mol Brain Res. (2005) 135:21–9. 10.1016/j.molbrainres.2004.11.006 15857665

[57] D'Cruz BJ Fertig KC Filiano AJ Hicks SD DeFranco DB Callaway CW. Hypothermic reperfusion after cardiac arrest augments brain-derived neurotrophic factor activation. J Cereb Blood Flow Metab. (2002) 22:843–51. 10.1097/00004647-200207000-00009 12142569

[58] Boris-Möller F Kamme F Wieloch T. The effect of hypothermia on the expression of neurotrophin mRNA in the hippocampus following transient cerebral ischemia in the rat. Brain Res Mol Brain Res. (1998) 63:163–73. 10.1016/S0169-328X(98)00286-1 9838092

[59] Zhao H Shimohata T Wang JQ Sun G Schaal DW Sapolsky RM. Akt contributes to neuroprotection by hypothermia against cerebral ischemia in rats. J Neurosci. (2005) 25:9794–806. 10.1523/JNEUROSCI.3163-05.2005 16237183 PMC6725740

[60] Lee SM Zhao H Maier CM Steinberg GK. The protective effect of early hypothermia on PTEN phosphorylation correlates with free radical inhibition in rat stroke. J Cereb Blood Flow Metab. (2009) 29:1589–600. 10.1038/jcbfm.2009.81 19553907 PMC3221613

[61] Ransohoff RM. Immunology: barrier to electrical storms. Nature. (2009) 457:155–6. 10.1038/457155a 19129836

[62] Deng H Han HS Cheng D Sun GH Yenari MA. Mild hypothermia inhibits inflammation after experimental stroke and brain inflammation. Stroke. (2003) 34:2495–501. 10.1161/01.STR.0000091269.67384.E7 12970518

[63] Perrone S Szabó M Bellieni CV Longini M Bangó M Kelen D. Whole body hypothermia and oxidative stress in babies with hypoxic-ischemic brain injury. Pediatr Neurol. (2010) 43:236–40. 10.1016/j.pediatrneurol.2010.05.009 20837300

[64] Wang Q Tang XN Yenari MA. The inflammatory response in stroke. J Neuroimmunol. (2007) 184:53–68. 10.1016/j.jneuroim.2006.11.014 17188755 PMC1868538

[65] Zheng Z Kim JY Ma H Lee JE Yenari MA. Anti-inflammatory effects of the 70 kDa heat shock protein in experimental stroke. J Cereb Blood Flow Metab. (2008) 28:53–63. 10.1038/sj.jcbfm.9600502 17473852

[66] Yenari MA Han HS. Influence of hypothermia on post-ischemic inflammation: role of nuclear factor kappa B (NFkappaB). Neurochem Int. (2006) 49:164–9. 10.1016/j.neuint.2006.03.016 16750872

[67] Choi JS Park J Suk K Moon C Park YK Han HS. Mild hypothermia attenuates intercellular adhesion molecule-1 induction via activation of extracellular signal-regulated kinase-1/2 in a focal cerebral ischemia model. Stroke Res Treat. (2011) 2011:846716. 10.4061/2011/846716 21716663 PMC3118291

[68] Tong G Krauss A Mochner J Wollersheim S Soltani P Berger F. Deep hypothermia therapy attenuates LPS-induced microglia neuroinflammation via the STAT3 pathway. Neuroscience. (2017) 358:201–10. 10.1016/j.neuroscience.2017.06.055 28687308

[69] Trendelenburg G. Acute neurodegeneration and the inflammasome: central processor for danger signals and the inflammatory response? J Cereb Blood Flow Metab. (2008) 28:867–81. 10.1038/sj.jcbfm.9600609 18212795

[70] Liu L Liu X Wang R Yan F Luo Y Chandra A. Mild focal hypothermia regulates the dynamic polarization of microglia after ischemic stroke in mice. Neurol Res. (2018) 40:508–15. 10.1080/01616412.2018.1454090 29619889

[71] Kim JY Kim JH Park J Beom JH Chung SP You JS. Targeted temperature management at 36 °C shows therapeutic effectiveness via alteration of microglial activation and polarization after ischemic stroke. Transl Stroke Res. (2022) 13:132–41. 10.1007/s12975-021-00910-8 33893993

[72] Lyden PD Lamb J Kothari S Toossi S Boitano P Rajput PS. Differential effects of hypothermia on neurovascular unit determine protective or toxic results: toward optimized therapeutic hypothermia. J Cereb Blood Flow Metab. (2019) 39:1693–709. 10.1177/0271678X18814614 30461327 PMC6727141

[73] Kurisu K Kim JY You J Yenari MA. Therapeutic hypothermia and neuroprotection in acute neurological disease. Curr Med Chem. (2019) 26:5430–55. 10.2174/0929867326666190506124836 31057103 PMC6913523

[74] Li X Li Y Zhang Z Bian Q Gao Z Zhang S. Mild hypothermia facilitates mitochondrial transfer from astrocytes to injured neurons during oxygen-glucose deprivation/reoxygenation. Neurosci Lett. (2021) 756:135940. 10.1016/j.neulet.2021.135940 33971244

[75] Wang L Wu L Duan Y Xu S Yang Y Yin J. Phenotype shifting in astrocytes account for benefits of intra-arterial selective cooling infusion in hypertensive rats of ischemic stroke. Neurotherapeutics. (2022) 19:386–98. 10.1007/s13311-022-01186-y 35044645 PMC9130426

[76] Mayer SA Kowalski RG Presciutti M Ostapkovich ND McGann E Fitzsimmons BF. Clinical trial of a novel surface cooling system for fever control in neurocritical care patients. Crit Care Med. (2004) 32:2508–15. 10.1097/01.CCM.0000147441.39670.37 15599159

[77] Kliegel A Losert H Sterz F Kliegel M Holzer M Uray T. Cold simple intravenous infusions preceding special endovascular cooling for faster induction of mild hypothermia after cardiac arrest–a feasibility study. Resuscitation. (2005) 64:347–51. 10.1016/j.resuscitation.2004.09.002 15733765

[78] Beom JH Kim JH Seo J Lee JH Chung YE Chung HS. Targeted temperature management at 33°C or 36°C induces equivalent myocardial protection by inhibiting HMGB1 release in myocardial ischemia/reperfusion injury. PLoS ONE. (2021) 16:e0246066. 10.1371/journal.pone.0246066 33503060 PMC7840046

[79] Oddo M Frangos S Maloney-Wilensky E Andrew Kofke W Le Roux PD Levine JM. Effect of shivering on brain tissue oxygenation during induced normothermia in patients with severe brain injury. Neurocrit Care. (2010) 12:10–6. 10.1007/s12028-009-9280-2 19821062

[80] Lotocki G de Rivero Vaccari JP Perez ER Sanchez-Molano J Furones-Alonso O Bramlett HM. Alterations in blood-brain barrier permeability to large and small molecules and leukocyte accumulation after traumatic brain injury: effects of post-traumatic hypothermia. J Neurotrauma. (2009) 26:1123–34. 10.1089/neu.2008.0802 19558276 PMC2848945

[81] Badjatia N. Hyperthermia and fever control in brain injury. Crit Care Med. (2009) 37:S250–7. 10.1097/CCM.0b013e3181aa5e8d 19535955

[82] Badjatia N. Fever control in the neuro-ICU: why, who, and when? Curr Opin Crit Care. (2009) 15:79–82. 10.1097/MCC.0b013e32832922e9 19578318

[83] Naito H Isotani E Callaway CW Hagioka S Morimoto N. Intracranial pressure increases during rewarming period after mild therapeutic hypothermia in postcardiac arrest patients. Ther Hypothermia Temp Manag. (2016) 6:189–93. 10.1089/ther.2016.0009 27213805

[84] Omileke D Pepperall D Bothwell SW Mackovski N Azarpeykan S Beard DJ. Ultra-Short duration hypothermia prevents intracranial pressure elevation following ischaemic stroke in rats. Front Neurol. (2021) 12:684353. 10.3389/fneur.2021.684353 34616350 PMC8488292

[85] Murtha LA McLeod DD McCann SK Pepperall D Chung S Levi CR. Short-duration hypothermia after ischemic stroke prevents delayed intracranial pressure rise. Int J Stroke. (2014) 9:553–9. 10.1111/ijs.12181 24025084

[86] Murtha LA Beard DJ Bourke JT Pepperall D McLeod DD Spratt NJ. Intracranial pressure elevation 24 h after ischemic stroke in aged rats is prevented by early, short hypothermia treatment. Front Aging Neurosci. (2016) 8:124. 10.3389/fnagi.2016.00124 27303291 PMC4882323

[87] Polderman KH Herold I. Therapeutic hypothermia and controlled normothermia in the intensive care unit: practical considerations, side effects, and cooling methods. Crit Care Med. (2009) 37:1101–20. 10.1097/CCM.0b013e3181962ad5 19237924

[88] Polderman KH Peerdeman SM Girbes AR. Hypophosphatemia and hypomagnesemia induced by cooling in patients with severe head injury. J Neurosurg. (2001) 94:697–705. 10.3171/jns.2001.94.5.0697 11354399

[89] Polderman KH. Mechanisms of action, physiological effects, and complications of hypothermia. Crit Care Med. (2009) 37:S186–202. 10.1097/CCM.0b013e3181aa5241 19535947

[90] Polderman KH. Application of therapeutic hypothermia in the intensive care unit. Opportunities and pitfalls of a promising treatment modality–Part 2: Practical aspects and side effects. Intensive Care Med. (2004) 30:757–69. 10.1007/s00134-003-2151-y 14767590

[91] Bergman R Braber A Adriaanse MA van Vugt R Tjan DH van Zanten AR. Haemodynamic consequences of mild therapeutic hypothermia after cardiac arrest. Eur J Anaesthesiol. (2010) 27:383–7. 10.1097/EJA.0b013e3283333a7d 19858724

[92] Callaway CW Donnino MW Fink EL Geocadin RG Golan E Kern KB. Part 8: post-cardiac arrest care: 2015 American Heart Association Guidelines update for cardiopulmonary resuscitation and emergency cardiovascular care. Circulation. (2015) 132:S465–82. 10.1161/CIR.0000000000000262 26472996 PMC4959439

[93] Kurisu K Yenari MA. Therapeutic hypothermia for ischemic stroke; pathophysiology and future promise. Neuropharmacology. (2018) 134:302–9. 10.1016/j.neuropharm.2017.08.025 28830757 PMC6919548

[94] van der Worp HB Sena ES Donnan GA Howells DW Macleod MR. Hypothermia in animal models of acute ischaemic stroke: a systematic review and meta-analysis. Brain. (2007) 130:3063–74. 10.1093/brain/awm083 17478443

[95] De Georgia MA Krieger DW Abou-Chebl A Devlin TG Jauss M Davis SM. Cooling for Acute Ischemic Brain Damage (COOL AID): a feasibility trial of endovascular cooling. Neurology. (2004) 63:312–7. 10.1212/01.WNL.0000129840.66938.75 15277626

[96] Krieger DW De Georgia MA Abou-Chebl A Andrefsky JC Sila CA Katzan IL. Cooling for acute ischemic brain damage (cool aid): an open pilot study of induced hypothermia in acute ischemic stroke. Stroke. (2001) 32:1847–54. 10.1161/01.STR.32.8.1847 11486115

[97] Lyden P Ernstrom K Raman R. Determinants of pneumonia risk during endovascular hypothermia. Ther Hypothermia Temp Manag. (2013) 3:24–7. 10.1089/ther.2012.0021 23667781 PMC3604784

[98] Ohta H Terao Y Shintani Y Kiyota Y. Therapeutic time window of post-ischemic mild hypothermia and the gene expression associated with the neuroprotection in rat focal cerebral ischemia. Neurosci Res. (2007) 57:424–33. 10.1016/j.neures.2006.12.002 17212971

[99] Markarian GZ Lee JH Stein DJ Hong SC Mild hypothermia: therapeutic window after experimental cerebral ischemia. Neurosurgery. (1996) 38:542–50. 10.1227/00006123-199603000-00024 8837807

[100] Eguchi Y Yamashita K Iwamoto T Ito H. Effects of brain temperature on calmodulin and microtubule-associated protein 2 immunoreactivity in the gerbil hippocampus following transient forebrain ischemia. J Neurotrauma. (1997) 14:109–18. 10.1089/neu.1997.14.109 9069442

[101] Neugebauer H Kollmar R Niesen WD Bösel J Schneider H Hobohm C. DEcompressive surgery Plus hypoTHermia for Space-Occupying Stroke (DEPTH-SOS): a protocol of a multicenter randomized controlled clinical trial and a literature review. Int J Stroke. (2013) 8:383–7. 10.1111/ijs.12086 23782729

[102] Neugebauer H Schneider H Bösel J Hobohm C Poli S Kollmar R. Outcomes of hypothermia in addition to decompressive hemicraniectomy in treatment of malignant middle cerebral artery stroke: a randomized clinical trial. JAMA Neurol. (2019) 76:571–9. 10.1001/jamaneurol.2018.4822 30657812 PMC6515837

[103] Li J Gu Y Li G Wang L Cheng X Wang M. The role of hypothermia in large hemispheric infarction: a systematic review and meta-analysis. Front Neurol. (2020) 11:549872. 10.3389/fneur.2020.549872 33192981 PMC7653189

[104] Basto FM Lyden P. Hypothermia in acute ischemic stroke therapy. Handb Clin Neurol. (2018) 157:823–37. 10.1016/B978-0-444-64074-1.00051-3 30459043

[105] Kuczynski AM Marzoughi S Al Sultan AS Colbourne F Menon BK van Es A. Therapeutic hypothermia in acute ischemic stroke-a systematic review and meta-analysis. Curr Neurol Neurosci Rep. (2020) 20:13. 10.1007/s11910-020-01029-3 32372297

[106] Hong JM Lee JS Song HJ Jeong HS Choi HA Lee K. Therapeutic hypothermia after recanalization in patients with acute ischemic stroke. Stroke. (2014) 45:134–40. 10.1161/STROKEAHA.113.003143 24203846

[107] Assis FR Narasimhan B Ziai W Tandri H. From systemic to selective brain cooling - methods in review. Brain Circ. (2019) 5:179–86. 10.4103/bc.bc_23_19 31950093 PMC6950511

[108] Choi JH Pile-Spellman J. Selective brain hypothermia. Handb Clin Neurol. (2018) 157:839–52. 10.1016/B978-0-444-64074-1.00052-5 30459044

[109] Wu C Zhao W An H Wu L Chen J Hussain M. Safety, feasibility, and potential efficacy of intraarterial selective cooling infusion for stroke patients treated with mechanical thrombectomy. J Cereb Blood Flow Metab. (2018) 38:2251–60. 10.1177/0271678X18790139 30019993 PMC6282221

[110] Tooley J Satas S Eagle R Silver IA Thoresen M. Significant selective head cooling can be maintained long-term after global hypoxia ischemia in newborn piglets. Pediatrics. (2002) 109:643–9. 10.1542/peds.109.4.643 11927709

[111] Qiu W Shen H Zhang Y Wang W Liu W Jiang Q. Noninvasive selective brain cooling by head and neck cooling is protective in severe traumatic brain injury. J Clin Neurosci. (2006) 13:995–1000. 10.1016/j.jocn.2006.02.027 17113984

[112] Nordberg P Taccone FS Truhlar A Forsberg S Hollenberg J Jonsson M. Effect of trans-nasal evaporative intra-arrest cooling on functional neurologic outcome in out-of-hospital cardiac arrest: the PRINCESS randomized clinical trial. JAMA. (2019) 321:1677–85. 10.1001/jama.2019.4149 31063573 PMC6506882

[113] Awad A Taccone FS Jonsson M Forsberg S Hollenberg J Truhlar A. Time to intra-arrest therapeutic hypothermia in out-of-hospital cardiac arrest patients and its association with neurologic outcome: a propensity matched sub-analysis of the PRINCESS trial. Intensive Care Med. (2020) 46:1361–70. 10.1007/s00134-020-06024-3 32514590 PMC7334260

[114] Huber C Huber M Ding Y. Evidence and opportunities of hypothermia in acute ischemic stroke: Clinical trials of systemic versus selective hypothermia. Brain Circ. (2019) 5:195–202. 10.4103/bc.bc_25_19 31950095 PMC6950508

[115] Duan H Huber M Ding JN Huber C Geng X. Local endovascular infusion and hypothermia in stroke therapy: a systematic review. Brain Circ. (2019) 5:68–73. 10.4103/bc.bc_9_19 31334359 PMC6611196

[116] Kammersgaard LP Rasmussen BH Jørgensen HS Reith J Weber U Olsen TS. Feasibility and safety of inducing modest hypothermia in awake patients with acute stroke through surface cooling: a case-control study: the Copenhagen Stroke Study. Stroke. (2000) 31:2251–6. 10.1161/01.STR.31.9.2251 10978060

[117] Kammersgaard LP Jørgensen HS Rungby JA Reith J Nakayama H Weber UJ. Admission body temperature predicts long-term mortality after acute stroke: the Copenhagen stroke study. Stroke. (2002) 33:1759–62. 10.1161/01.STR.0000019910.90280.F1 12105348

[118] Ding Y Li J Luan X Lai Q McAllister JP 2nd. Local saline infusion into ischemic territory induces regional brain cooling and neuroprotection in rats with transient middle cerebral artery occlusion. Neurosurgery. (2004) 54:956–64. 10.1227/01.NEU.0000114513.96704.29 15046664

[119] Ding Y Yao B Zhou Y Park H McAllister JP. 2nd, Diaz FG Prereperfusion flushing of ischemic territory: a therapeutic study in which histological and behavioral assessments were used to measure ischemia-reperfusion injury in rats with stroke. J Neurosurg. (2002) 96:310–9. 10.3171/jns.2002.96.2.0310 11838805

[120] Ji Y Hu Y Wu Y Ji Z Song W Wang S. Therapeutic time window of hypothermia is broader than cerebral artery flushing in carotid saline infusion after transient focal ischemic stroke in rats. Neurol Res. (2012) 34:657–63. 10.1179/1743132812Y.0000000061 22709718

[121] Ji YB Wu YM Ji Z Song W Xu SY Wang Y. Interrupted intracarotid artery cold saline infusion as an alternative method for neuroprotection after ischemic stroke. Neurosurg Focus. (2012) 33:E10. 10.3171/2012.5.FOCUS1215 22746227

[122] Lassen NA. The luxury-perfusion syndrome and its possible relation to acute metabolic acidosis localised within the brain. Lancet. (1966) 2:1113–5. 10.1016/S0140-6736(66)92199-4 4162534

[123] Choi JH Marshall RS Neimark MA Konstas AA Lin E Chiang YT. Selective brain cooling with endovascular intracarotid infusion of cold saline: a pilot feasibility study. AJNR Am J Neuroradiol. (2010) 31:928–34. 10.3174/ajnr.A1961 20053807 PMC7964178

[124] Chen J Liu L Zhang H Geng X Jiao L Li G. Endovascular hypothermia in acute ischemic stroke: pilot study of selective intra-arterial cold saline infusion. Stroke. (2016) 47:1933–5. 10.1161/STROKEAHA.116.012727 27197848 PMC4927369

[125] Choi JH Poli S Chen M Nguyen TN Saver JL Matouk C. Selective brain hypothermia in acute ischemic stroke: reperfusion without reperfusion injury. Front Neurol. (2020) 11:594289. 10.3389/fneur.2020.594289 33281733 PMC7691595

[126] Rha JH Saver JL. The impact of recanalization on ischemic stroke outcome: a meta-analysis. Stroke. (2007) 38:967–73. 10.1161/01.STR.0000258112.14918.24 17272772

[127] Fazel Bakhsheshi M Keenliside L Lee TY. A novel selective cooling system for the brain: feasibility study in Rabbits vs piglets. Intensive Care Med Exp. (2018) 6:45. 10.1186/s40635-018-0211-4 30387029 PMC6212374

[128] Madden LK Hill M May TL Human T Guanci MM Jacobi J. The Implementation of targeted temperature management: an evidence-based guideline from the neurocritical care society. Neurocrit Care. (2017) 27:468–87. 10.1007/s12028-017-0469-5 29038971

[129] Marehbian J Greer DM. Normothermia and stroke. Curr Treat Options Neurol. (2017) 19:4. 10.1007/s11940-017-0437-6 28243991

[130] Zeiner A Holzer M Sterz F Schörkhuber W Eisenburger P Havel C. Hyperthermia after cardiac arrest is associated with an unfavorable neurologic outcome. Arch Intern Med. (2001) 161:2007–12. 10.1001/archinte.161.16.2007 11525703

[131] Taccone FS Lascarrou JB Skrifvars MB. Targeted temperature management and cardiac arrest after the TTM-2 study. Crit Care. (2021) 25:275. 10.1186/s13054-021-03718-y 34348777 PMC8336232

[132] Dankiewicz J Cronberg T Lilja G Jakobsen JC Levin H Ullén S. Hypothermia versus Normothermia after Out-of-Hospital Cardiac Arrest. N Engl J Med. (2021) 384:2283–94. 10.1056/NEJMoa2100591 34133859

[133] Connolly ES Jr Rabinstein AA Carhuapoma JR Derdeyn CP Dion J Higashida RT. Guidelines for the management of aneurysmal subarachnoid hemorrhage: a guideline for healthcare professionals from the American Heart Association/American Stroke Association. Stroke. (2012) 43:1711–37. 10.1161/STR.0b013e3182587839 22556195

[134] Piironen K Tiainen M Mustanoja S Kaukonen KM Meretoja A Tatlisumak T. Mild hypothermia after intravenous thrombolysis in patients with acute stroke: a randomized controlled trial. Stroke. (2014) 45:486–91. 10.1161/STROKEAHA.113.003180 24436240

